# Heat Shock Protein 22 (Hsp22) Regulates Oxidative Phosphorylation upon Its Mitochondrial Translocation with the Inducible Nitric Oxide Synthase in Mammalian Heart

**DOI:** 10.1371/journal.pone.0119537

**Published:** 2015-03-06

**Authors:** Eman Rashed, Paulo Lizano, Huacheng Dai, Andrew Thomas, Carolyn K. Suzuki, Christophe Depre, Hongyu Qiu

**Affiliations:** 1 Department of Cell Biology and Molecular Medicine, New Jersey Medical School, Rutgers, The State University of New Jersey, New Brunswick, New Jersey, United States of America; 2 Department of Pharmacology and Physiology, New Jersey Medical School, Rutgers, The State University of New Jersey, New Brunswick, New Jersey, United States of America; 3 Department of Microbiology, Biochemistry and Molecular Genetics, New Jersey Medical School, Rutgers, The State University of New Jersey, New Brunswick, New Jersey, United States of America; 4 Department of Basic Science, Division of Physiology, School of Medicine, Loma Linda University, Loma Linda, California, United States of America

## Abstract

**Objectives:**

Stress-inducible heat shock protein 22 (Hsp22) confers protection against ischemia through induction of the inducible isoform of nitric oxide synthase (iNOS). Hsp22 overexpression *in vivo* stimulates cardiac mitochondrial respiration, whereas Hsp22 deletion *in vivo* significantly reduces respiration. We hypothesized that Hsp22-mediated regulation of mitochondrial function is dependent upon its mitochondrial translocation together with iNOS.

**Methods and Results:**

Adenoviruses harboring either the full coding sequence of Hsp22 (Ad-WT-Hsp22) or a mutant lacking a N-terminal 20 amino acid putative mitochondrial localization sequence (Ad-N20-Hsp22) were generated, and infected in rat neonatal cardiomyocytes. Compared to β-Gal control, WT-Hsp22 accumulated in mitochondria by 2.5 fold (P<0.05) and increased oxygen consumption rates by 2-fold (P<0.01). This latter effect was abolished upon addition of the selective iNOS inhibitor, 1400W. Ad-WT-Hsp22 significantly increased global iNOS expression by about 2.5-fold (P<0.01), and also increased iNOS mitochondrial localization by 4.5 fold vs β-gal (P<0.05). Upon comparable overexpression, the N20-Hsp22 mutant did not show significant mitochondrial translocation or stimulation of mitochondrial respiration. Moreover, although N20-Hsp22 did increase global iNOS expression by 4.6-fold, it did not promote iNOS mitochondrial translocation.

**Conclusion:**

Translocation of both Hsp22 and iNOS to the mitochondria is necessary for Hsp22-mediated stimulation of oxidative phosphorylation.

## Introduction

The heart depends on oxidative phosphorylation to supply the large amount of ATP required for its continuous contractile activity[1]. Mitochondrial dysfunction due to decreased supply of oxygen and substrates, such as that occurring during ischemia, leads to impaired ATP generation and structural alterations of the respiratory chain, eventually resulting in cell death by both apoptosis and necrosis[2].

The heat shock protein 22/H11 Kinase (Hsp22) is a stress-inducible protein responsive to various conditions of myocardial stress, including ischemia [3,4]. Cardiac-specific overexpression of Hsp22 in a transgenic (TG) mouse provides protection against myocardial ischemia that is equally powerful to ischemic preconditioning [5] through the induction of the inducible isoform of nitric oxide synthase (iNOS) [5], the effector of the second window of ischemic preconditioning [6]. Accordingly, inhibition of iNOS abolishes the cardioprotection conferred by Hsp22 [7].

We characterized previously the subcellular distribution of Hsp22 in the heart, and showed that it is located in the mitochondrial, as well as in the nuclear and cytosolic fractions [8]. In addition, it has been shown in *Drosophila* that Hsp22 localization in mitochondria is due to a translocation mechanism that depends on its N-terminal domain [9]. The biological function of Hsp22 inside mammalian mitochondria remains unknown; however we showed in the TG mouse model that increased Hsp22 expression *in vivo* stimulates mitochondrial oxidative phosphorylation, whereas its deletion in a knockout model has the opposite effect [10]. In addition, the TG mouse is characterized by an inhibition of the mitochondrial pathway of apoptosis [5]. These observations support the hypothesis that mitochondrial localization of Hsp22 might promote both cardiac cell survival and oxidative metabolism. The main purpose of our study was to interrogate the physiological consequence of this mechanism on mitochondrial respiration.

Although our previous studies showed that cardioprotection conferred by Hsp22 is iNOS-dependent, the role played by iNOS in the mitochondrial respiration is still relatively unknown. We also hypothesized that mitochondrial functions of Hsp22 could be mediated by iNOS. Therefore, in the present study, we tested whether mitochondrial localization of Hsp22 in mammalian heart promotes mitochondrial respiration, and whether this process involves iNOS.

## Materials and Methods

### Animal model

Three-month old male FVB wild type (WT) and Hsp22 transgenic (TG) mice were used. A cardiac specific Hsp22 TG mouse was generated as described previously [11]. The Hsp22 protein in the transgene is hemagglutinin-tagged, which confers a molecular weight of 27 kDa as compared to 25 kDa for the WT protein. Animals were euthanized with 100 mg/kg pentobarbital. The investigation conforms to the Guide for the Care and Use of Laboratory Animals published by the US National Institutes of Health (NIH Publication No. 85-23, revised 1996), and with the approval of the IACUC committee at New Jersey Medical School, Rutgers, The State University of New Jersey (IACUC# 11025 and #11024).

### Generation of a mutant Hsp22 adenovirus

Two mutant adenoviruses were generated, in which the last 20 amino acids at either the N-terminus (Ad-N20-Hsp22) or the C-terminus (Ad-C20-Hsp22) were deleted. A green fluorescent protein (GFP) coding sequence was fused at the C-terminus of each mutant. The sequences were ligated downstream from the CMV promoter of the AdEasy XL adenoviral vector system (Agilent, Santa Clara, CA), followed by homologous recombination with the adenoviral backbone plasmid [11]. The adenoviruses harboring the full length Hsp22 coding sequence (Ad-WT-Hsp22) or the β-galactosidase (β-Gal) control were described before [11,12]. The recombinant adenoviruses were propagated in HEK 293 cells.

### Culture of rat neonatal cardiac myocytes

Rat neonatal ventricular cardiac myocytes (RNCMs) were prepared from Sprague-Dawley rat pups (Charles River Laboratories, Wilmington, MA) as described previously [11,13,14]. Neonatal rats were sterilized with 70% ethanol and sacrificed by decapitation. Myocytes were dispersed with 0.1% collagenase type IV (Worthington Biochem, Lakewood, NJ), 0.1% trypsin (GIBCO, Grand Island, NY) and 15 μg/mL DNase I (Sigma-Aldrich, St. Louis, MO). Cell suspensions were applied on a discontinuous Percoll gradient, and the myocyte layer was collected. Cells were cultured in medium containing Dulbecco’s Modified Eagle Medium (DMEM)/F12 (1:1) (Sigma-Aldrich, St. Louis, MO) for 24 hours. Myocytes were infected for 48 hours after 24 hours of serum-free starvation. Inhibition of iNOS was initiated 24 hours before the collection of the cells upon addition of 100 μM 1400W (Sigma-Aldrich, St. Louis, MO).

### Protein extraction and subcellular fractions

Tissues were homogenized at 4°C in a buffer supplemented with protease and phosphatase inhibitors [5,15], and centrifuged at 12,000*g* for 20 min. Proteins were denatured by boiling, resolved on SDS-PAGE gels and then transferred onto a nitrocellulose membrane. Primary antibodies were incubated overnight and after incubation with the secondary antibody, detection was performed by chemiluminescence (Dupont/NEN, Boston, MA), and quantified by densitometry. Subcellular fractions were prepared as described previously [15]. After an initial spin at 100*g*, the nuclear fraction was pelleted at low-speed centrifugation (500*g*, 10 minutes). The supernatant was further centrifuged (10,000*g*, 10 minutes) to pellet the mitochondrial fraction. The resulting supernatant was ultracentrifuged (100,000*g*, 90 minutes) to obtain the cytosolic fraction (supernatant) and a microsomal fraction (pellet). Pellets were washed and re-suspended in 150 mM NaCl, 1% NP40, 0.5% deoxycholate, 0.1% SDS, 50 mM Tris (pH 8.0). Fraction purity was verified by western blotting, using glyceraldehyde 3-phosphate dehydrogenase (GAPDH, cytosol), lamin A/C (nucleus), cytochrome oxidase IV (COX IV, mitochondria), and plasma membrane calcium ATPase (membranes) as described previously [8]. Targeted proteins including Hsp22, Stat3 and iNOS were detected by western blotting as described previously [8,10].

### Mitochondrial sub-fractionation

Two techniques were used to sub-fractionate the mitochondria obtained by cell fractionation. First, isolated mitochondria were incubated with 2% digitonin for 20 minutes, and spun at 9,600*g* for 15 minutes, which yielded the mitoplasts in the pellet, and the outer mitochondrial membrane (OM) together with the inter-membrane space (IMS) in the supernatant [9,16,17]. Second, mitochondria were incubated with 2% Nonidet P-40 for 15 minutes and spun at 18,000*g* for 40 minutes to yield a soluble component including the matrix, IMS and an insoluble fraction composed of mitochondrial inner (IM) and outer membranes (OM) [9]. The purity of each mitochondrial sub-fraction was verified by western blotting using specific antibodies: COX IV (1:1000, rabbit polyclonal antibodies, Cell Signaling, Danvers, MA) for IM, voltage-dependent anion-selective channel protein 1 [VDAC] (1:1000, rabbit polyclonal antibodies, Cell Signaling, Danvers, MA) for OM, and Grp75 (1:500, Rabbit polyclonal antibody, Abcam, Cambridge, MA) for the matrix.

### Oxygen consumption assay

**Clark electrode**. Mitochondrial oxygen consumption rates (OCR) were measured in intact RNCMs with a Clark-type electrode as described previously [18,19]. Briefly, RNCMs infected with Ad-β-Gal, Ad-WT-Hsp22 or Ad-N20-Hsp22 were collected and suspended in extracellular buffer as described[18] Glucose was used as a substrate. Oxygen concentration was measured at 30°C with a Clark-type electrode fitted to a 50 μl water-jacketed reaction chamber. OCR was determined after addition of 6 μM oligomycin or 5 μM carbonyl cyanide 4-(trifluoromethoxy) phenylhydrazone (FCCP). The ratio of FCCP-stimulated to oligomycin-inhibited OCR in the myocytes was also calculated.

### Immunofluorescence

Cardiac myocytes were cultured to confluence on a 4-chamber slide. The cells were washed with PBS, fixed in methanol at −20°C for 10 min, blocked with 5% bovine serum albumin for 1h, and incubated with primary antibody. After washing, cells were incubated with a fluorescein-labeled secondary antibody and mounted in a Vecto 4’-6-diamino-2-phenylindole (DAPI) medium for observation at x40. Mitochondria were detected using the MitoTracker Red (Life Technologies, Carlsbad, CA).

### Measurement of iNOS activity

Cell lysates were incubated in 20 mM Tris–HCl (pH 8.0), 2 mM NADPH, 2 mM L-arginine and 10 mM FAD for 3 h at room temperature. NO production was measured from nitrite levels based on the Griess reaction [20] (Nitric Oxide Assay Kit, Oxford Biomedical Research, Oxford, UK). The absorbance values were determined at 540 nm using a microtiter plate reader. Nitrate was reduced to nitrite by incubation with 0.1 U/ml nitrate reductase, 0.1 mM NADPH and 5 mM FAD. The reaction was stopped by the addition of 10 U/ml lactate dehydrogenase and 10 mM pyruvate. Sodium nitrite was used as a standard, and lysis buffer as a blank. Nitrite value of the control test (without NADPH/ L-arginine) was subtracted from the experimental values [21,22].

### Apoptosis assays

Apoptosis was induced upon incubation of RCNMs with 1μM chelerythrine (Sigma Aldrich, St. Louis, MO) for 4 hours and was measured, both by enzyme-linked colorimetric assay and by TUNEL staining as described previously[8].

### Statistical analysis

Results are the mean ± SEM for the number of samples indicated in the Figure legends. A one-way ANOVA was used and Student-Newman-Keuls post hoc correction was applied for multi-group comparison. A value of P<0.05 was considered significant.

## Results

### Hsp22 is predominantly located to the mitochondrial inner membrane

**Fig 1:**
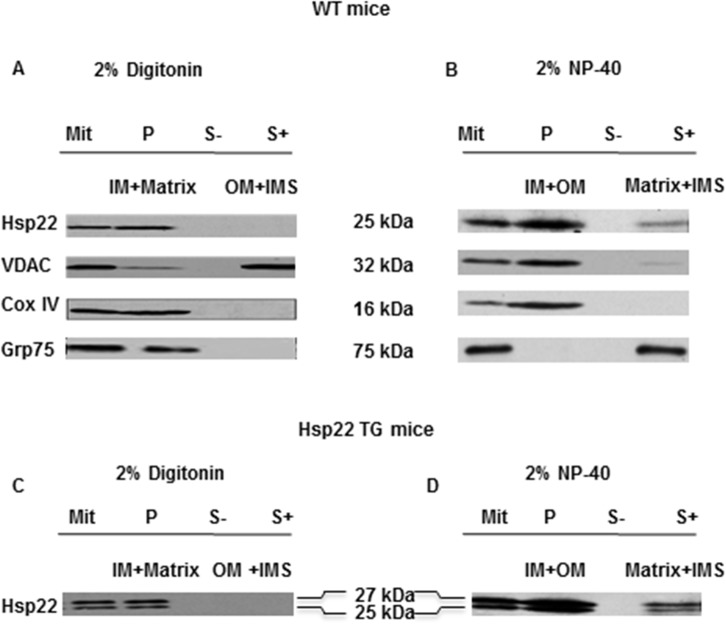
Sub-mitochondrial localization of Hsp22 in mouse heart. Mitochondrial sub-fractionation was performed on WT (A and B) and TG mouse hearts (C and D) after treatment with digitonin (A and C) and NP-40 (B and D). In each panel, from left to right: Mit: untreated total mitochondria (positive control); P: pellet obtained from detergent-treated mitochondria; S-: supernatant obtained after centrifugation of untreated mitochondria (negative control); S+: supernatant obtained after treatment. In panels A and C, digitonin treatment shows that Hsp22 is distributed in the pellet (P) containing the IM and matrix but not in the supernatant (S+) containing the OM and IMS. In panels B and D, NP-40 treatment shows that Hsp22 is predominantly located in the pellet (P) containing the IM and OM, and to a low extent in the supernatant (S+) containing the matrix and IMS. Markers include VDAC (OM), COX IV (IM), and Grp75 (matrix). The figure shows one representative example of n = 4 per group.

We previously showed that Hsp22 is expressed in mitochondria from mouse heart [8]. To elucidate the potential role of Hsp22 in the mitochondria, we first characterized its sub-mitochondrial localization using two different fractionation techniques described in the Methods. As shown in Fig. 1 A and B, the digitonin digestion method showed that Hsp22 was only detected in the pellet sub-fraction containing the IM and matrix, but not in the fraction containing the OM and IMS. The NP-40 digestion showed that Hsp22 was located primarily within the membrane fraction, whereas only a residual amount was found in the matrix and IMS. Taken together, these results demonstrate that, in the mammalian heart, mitochondrial Hsp22 is predominantly associated with the IM. We repeated this experiment in the TG model of cardiac-specific Hsp22 overexpression. The results shown in Fig. 1 C and D are consistent with the data obtained in the WT mouse, indicating that over-expression of the protein does not affect its sub-mitochondrial localization. This observation is important in order to validate the experiments of overexpression shown below in RNCMs.

### N-terminus deletion prevents the mitochondrial localization of Hsp22

**Fig 2:**
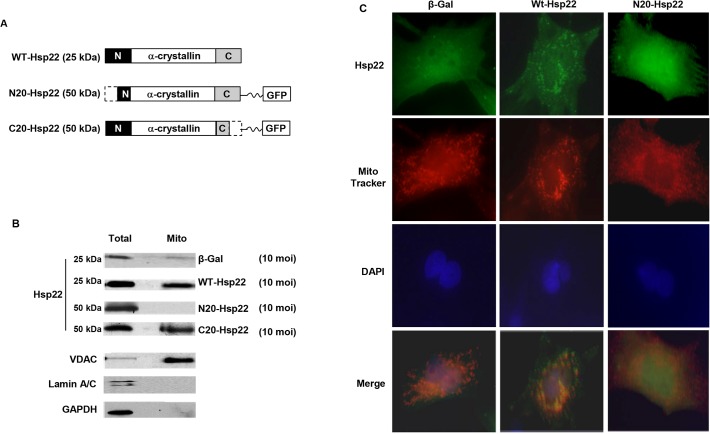
N-terminal deletion of the Hsp22 coding sequence prevents its mitochondrial localization. A. Hsp22 mutants were generated by deletion of 20 amino acids at either the N-terminus (Ad-N20-Hsp22) or C-terminus (Ad-C20-Hsp22), respectively, from the Hsp22 WT coding sequence (Ad-WT-Hsp22). B. Examples of the ratio of Hsp22 between the mitochondrial fraction and the total protein extract for the corresponding protein extracted from cardiac myocytes treated with β-Gal, WT-Hsp22, N20-Hsp22 and C20-Hsp22 adenoviruses (10 moi). Markers include VDAC (mitochondria), lamin A/C (nucleus), and GAPDH (total extracts). C. Immunofluorescence on myocytes infected with 10 moi β-Gals, Ad-WT-Hsp22 and Ad-N20-Hsp22. Staining is performed with Hsp22 or GFP antibodies (green), Mitotracker (red) and nuclear DAPI counterstaining (blue), followed by merging of the three signals.

To determine the domain responsible for the mitochondrial localization of Hsp22 in mammalian heart, an adenovirus harboring a mutant lacking a 20 amino acid putative N-terminal mitochondrial localization sequence [9] was generated (Ad-N20-Hsp22), and infected in rat neonatal cardiomyocytes. An adenovirus harboring the complete coding sequence of Hsp22 (Ad-WT-Hsp22) was used as a positive control, whereas an adenovirus harboring the sequence of β-galactosidase (β-Gal) was used as a negative control (Fig. 2 A). Ad-WT-Hsp22 and Ad-N20-Hsp22 were infected at 10 moi in RNCMs. Overexpression of the corresponding protein was comparable between WT-Hsp22 and N20-Hsp22-adenoviruses (Fig. 2 B). Mitochondria were further separated by sub-cellular fractionation. Although cells over-expressing Ad-WT-Hsp22 exhibited a significant increase (2.5 fold) in mitochondrial Hsp22 protein as compared to the β-Gal control, such accumulation was not observed in myocytes treated with Ad-N20-Hsp22 (Fig. 2 B). In comparison, myocytes treated with Ad-N20-Hsp22 actually showed a loss of mitochondrial Hsp22 localization (Fig. 2 B). We also generated another mutant adenovirus in which the last 20 amino acids at the C-terminus (Ad-C20-Hsp22) were deleted. A green fluorescent protein (GFP) coding sequence was fused at the C-terminus of mutant as the same as Ad-N20-Hsp22. Cells over-expressing Ad-C20-Hsp22 exhibited a significant increase in mitochondrial Hsp22 protein as compared to the β-Gal control which is similar to Ad-WT-Hsp2, indicating that it is the deletion of the 20 amino acid putative N-terminal mitochondrial localization sequence and not the GFP fusion protein that is responsible for the failure of Ad-N20-Hsp22 mutant protein to be taken up into the mitochondria (Fig. 2A and B).

Immunofluorescence was performed on RNCMs infected with 10 moi of the respective adenoviruses for 48 hours. Myocytes infected with Ad-WT-Hsp22 showed an increased Hsp22 expression in a punctate pattern, which superimposed with the MitoTracker signal, indicating a mitochondrial localization (Fig. 2 C). Myocytes infected with Ad-N20-Hsp22 showed an increase in exogenous, GFP-tagged Hsp22, which did not superimpose with Mitotracker (red), indicating a loss of the mitochondrial localization of the mutant Hsp22 (Fig. 2 C).

### N-terminus deletion does not affect function of Hsp22 protein on gene expression

**Fig 3:**
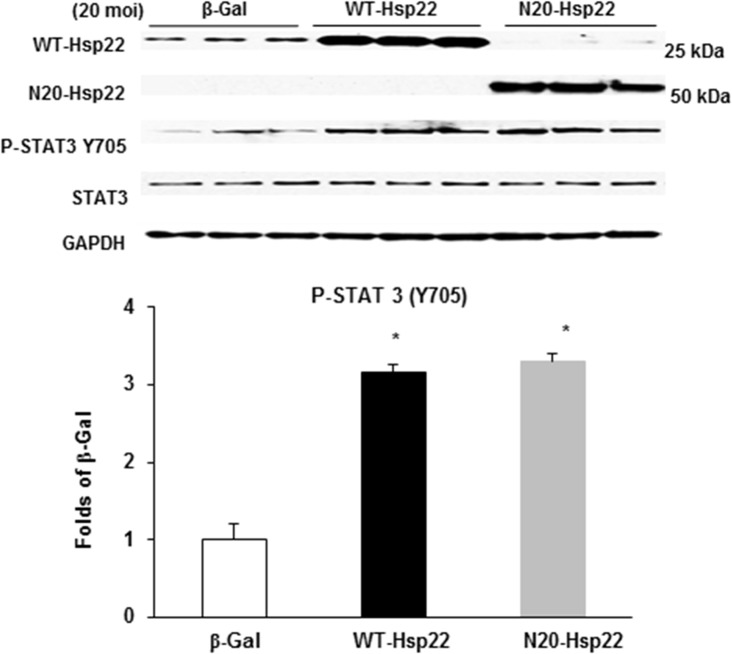
Regulation of gene expression of Hsp22 mutant proteins in cardiac myocytes. Immunoblotting of P-STAT3 (Y705) in myocytes treated with the β-Gal, WT-Hsp22 or N20-Hsp22 adenoviruses (20 moi). * P<0.05 vs β-Gal, n = 6 per group.

We showed before that Hsp22 over-expression regulates gene expression through the regulation of specific transcription factors, including STAT3 [10]. Therefore, we investigated whether the N-terminal mutant Hsp22 protein preserved its biological function on transcriptional regulation. Ad-WT-Hsp22 and Ad-N20-Hsp22 were infected at 20 moi in RNCMs. Y705 phosphorylation of the transcription factor STAT3, a target through which Hsp22 affects gene expression [10], was used as a control to show the activation of the STAT3 protein. As shown in Fig. 3, upon a comparable amount of Hsp22 overexpression, myocytes infected with either Ad-WT-Hsp22 or Ad-N20-Hsp22 exhibited increased Y705 phosphorylation of STAT3 by about 3.0 fold vs β-Gal control (P<0.05). Therefore, the truncated Hsp22 protein retains its native function and activity on transcriptional regulation.

### Mitochondrial localization of Hsp22 is necessary for cytoprotection conferred by Hsp22

**Fig 4:**
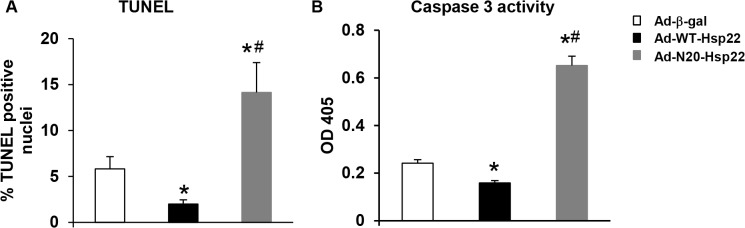
Mitochondrial localization of Hsp22 is necessary for cellular protection in cardiac myocytes. Apoptosis measured by TUNEL and caspase-3 activity in RNCMs infected with the β-Gal control, Ad-WT-Hsp22 or Ad-N20-Hsp22 with chelerythrine. *, P<0.05 versus β-Gal; #, P<0.05 vs Ad-WT-Hsp22. n = 5 per group.

We have shown previously that overexpression of Hsp22 promotes cell survival by inhibiting cardiomyocyte apoptosis. We next tested whether the N-terminal mutant of Hsp22 influences its cytoprotection against apoptosis. Myocytes were infected with an adenovirus harboring the Ad-WT-Hsp22 or Ad-N20-Hsp22 at 10 moi for 48 hours, and compared with β-Gal. Chelerythrine was subsequently added to induce cell apoptosis. Apoptosis was first measured by TUNEL. As shown in Fig. 4 A, over-expression of WT-Hsp22 reduced chelerythrine-induced apoptosis by 60% (P<0.05) compared to β-Gal. No protection was observed in myocytes infected with the Ad-N20-Hsp22, which actually induced a 2.5-fold increase in apoptosis compared with the β-Gal control. A caspase-3 colorimetric assay confirmed the results found by TUNEL. Again, while overexpression of Hsp22 markedly reduced chelerythrine-induced caspase 3 activation compared to the β-Gal group, overexpression of Ad-N20-Hsp22 significantly increased caspase 3 activity by 3-fold (Fig. 4 B).

### N-terminus deletion abolishes Hsp22-stimulated oxidative phosphorylation

**Fig 5:**
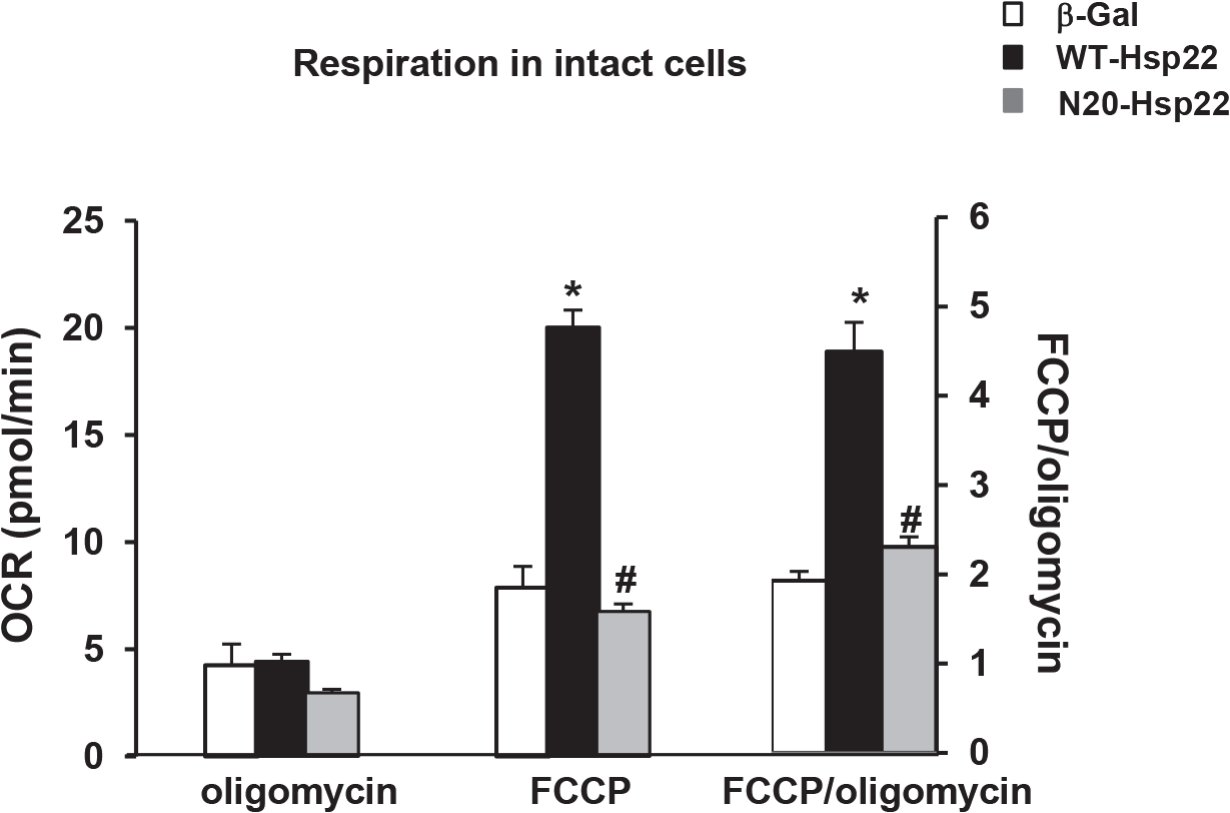
Effect of N-terminus mutant Hsp22 on mitochondrial respiration in intact neonatal rat cardiac myocytes. Mitochondrial respiration measured by Clark electrode in intact myocytes transfected with Ad-WT-Hsp22 or Ad-N20-Hsp22 compared to β-Gal. Mitochondrial respiration is presented on the left Y axis as OCR after addition of oligomycin and OCR after addition of FCCP, and, on the right Y axis, as the ratio of both rates (FCCP/oligomycin). *, P<0.05 versus β-Gal; #, P<0.05 vs Ad-WT-Hsp22. n = 5 per group.

Since our previous results *in vivo* showed that manipulation of Hsp22 expression in the heart stimulates mitochondrial respiration [10], we tested next whether preventing Hsp22 mitochondrial localization with the N-terminal mutant blocks the effect of the protein on mitochondria. A Clark-type oxygen electrode was used to measure the oxygen consumption rate (OCR) in intact RNCMs transfected with Ad-WT-Hsp22 or Ad-N20-Hsp22, and compared to β-Gal (Fig. 5). The rate of mitochondrial respiration was measured under basal (pseudo State 4) conditions in the presence of oligomycin to block the F_1_F_o_ ATP synthase, followed by addition of the uncoupler FCCP. In intact cells, the difference between OCR in the presence of oligomycin [18] and in the presence of rotenone, represents proton leak across the inner mitochondrial membrane, while OCR in the presence of FCCP represents the maximal respiratory capacity, and indicates the potential of the cell to maintain its energy balance in stress conditions [18]. Therefore, the ratio of these two rates represents the "spare" respiratory capacity of cells to respond to conditions of increased energy demand.

As shown in Fig. 5, there was no significant difference in the leak rate in the three treatment groups measured in the presence of oligomycin. The maximal OCR after addition of FCCP increased by about 2.5-fold in myocytes treated with Ad-WT-Hsp22 (10 moi) compared to the β-Gal control (P<0.05), but this was not observed with the mutant Ad-N20-Hsp22. Consequently, the FCCP/oligomycin OCR ratio increased significantly (P<0.05) in myocytes treated with Ad-WT-Hsp22, but not in myocytes infected with Ad-N20-Hsp22 (Fig. 5). These data show that Hsp22 overexpression does not uncouple the mitochondria, but rather it increases its maximally stimulated respiration, which was not observed upon overexpression of the Ad-N20-Hsp22 mutant. Thus, the effect of Hsp22 on mitochondrial respiration is dependent upon its translocation to the mitochondria via its N-terminus.

### Hsp22 stimulates mitochondrial respiration through iNOS

**Fig 6:**
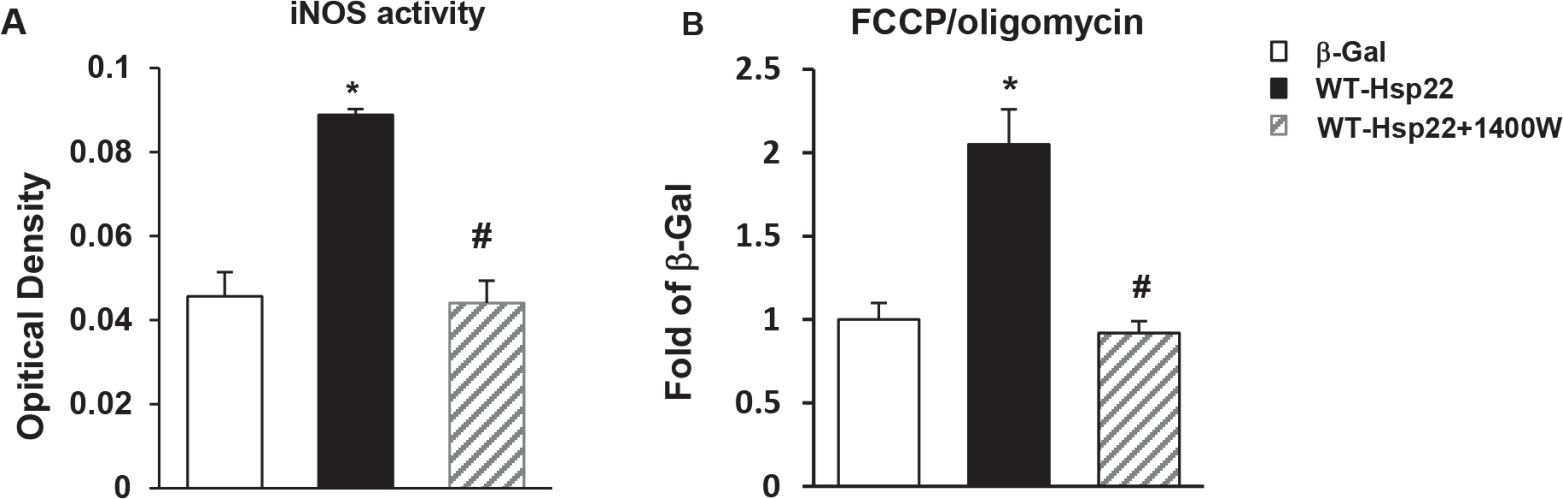
Hsp22 stimulates mitochondrial respiration through iNOS. A. iNOS activity in myocytes transfected with Ad-WT-Hsp22 in presence or in absence of 1400W, compared to β-Gal. Optical density represents the measured absorbance at 540 nm. *, P<0.05 versus β-Gal; #, P<0.05 vs Ad-WT-Hsp22 without 1400W. n = 5 per group. B. Mitochondrial respiration measured by Clark electrode in intact myocytes transfected with Ad-WT-Hsp22 in presence or in absence of the iNOS inhibitor 1400W, and compared to β-Gal control, as presented by the FCCP/oligomycin ratio. *, P<0.05 versus β-Gal; #, P<0.05 vs Ad-WT-Hsp22 without 1400W. n = 4 per group.

Our previous studies showed that iNOS is central to the mechanism of cardioprotection by Hsp22 [7], therefore we tested whether the effect of Hsp22 on mitochondrial respiration could be mediated by iNOS. Myocytes were transfected with Ad-WT-Hsp22 for 24 hours, in the absence or presence of the selective iNOS inhibitor 1400W [23]. Mitochondrial respiration was measured in these conditions by a Clark-type oxygen electrode (see Methods). As shown in Fig. 6 A, Hsp22 overexpression increased iNOS activity by approximately 2-fold, which was abolished upon addition of 1400W (Fig. 6 A). Consistent with the studies described above, myocytes treated with Ad-WT-Hsp22 exhibited a 2-fold increase in OCR (P<0.05) compared to β-Gal, as represented by the FCCP/oligomycin ratio. This stimulation of mitochondrial oxygen consumption by Ad-WT-Hsp22 was also abolished by 1400W (Fig. 6 B). Therefore, the effect of Hsp22 on mitochondrial respiration is shown to be dependent upon iNOS. This is consistent with the data presented in Fig. 5, showing that overexpression of Ad-WT-Hsp22 in myocytes exhibited an increase in mitochondrial respiration compared to β-Gal, which was abolished upon the over-expression of Ad-N20-Hsp22.

### The N-terminal mutant Hsp22 blocks the translocation of iNOS into mitochondria

We showed above that the N-terminal deletion of Hsp22 prevented its translocation into mitochondria (Fig. 2), and abolished Hsp22-stimulated mitochondrial respiration (Fig. 5). The later observation is consistent with the effect of the iNOS inhibitor on Hsp22-stimulated mitochondrial respiration as shown in Fig. 6 B. Therefore, we tested whether the mitochondrial translocation of Hsp22 affects the expression and/or regulation of iNOS in the mitochondria.

**Fig 7:**
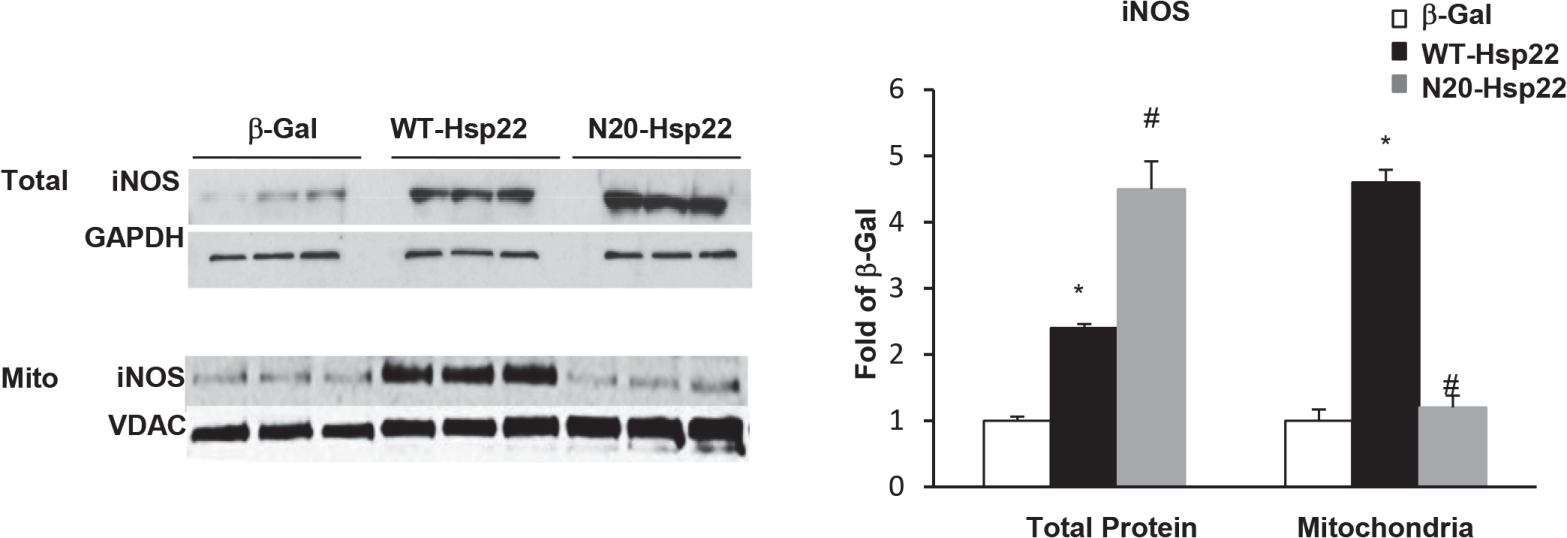
Mitochondrial translocation of iNOS by Hsp22. iNOS expression in total cell lysates and mitochondrial fractions from myocytes treated with Ad-WT-Hsp22 or Ad-N20-Hsp22 compared to β-Gal. *, P<0.05 vs β-Gal; #, P<0.05 vs Ad-WT-Hsp22. n = 6 per group.

We first tested whether the Ad-N20-Hsp22 mutant affects iNOS expression at a global, cellular level. RNCMs were infected with 20 moi of Ad-WT-Hsp22 or Ad-N20-Hsp22, and compared to β-Gal control. Immunoblotting for iNOS was performed using total cell lysates. Compared to β-Gal control, myocytes infected with Ad-WT-Hsp22 exhibited a significant 2.5-fold increase in global iNOS expression. Myocytes treated with Ad-N20-Hsp22 exhibited a significant global increase of iNOS expression by 4.6-fold in total cellular lysate (Fig. 7), indicating that the mutant Hsp22 did not impair the regulation of Hsp22 on iNOS gene expression. This result is consistent with the observation that the mutant Hsp22 maintains the activation of STAT3 (Fig. 3), a transcription factor known to up-regulate iNOS expression [6,10].

Next, we tested whether Hsp22 affects the mitochondrial translocation of iNOS. As shown in Fig. 7, iNOS expression in the mitochondrial fraction was significantly increased by 4.5-fold in myocytes infected with Ad-WT-Hsp22 (P<0.05 versus β-Gal) but this increase was not observed in mitochondria of myocytes treated with Ad-N20-Hsp22 (Fig. 7). These data indicate that the mutant Hsp22 protein prevented mitochondrial localization of iNOS despite the substantial increase in total iNOS protein expression. Together with the data shown in Fig. 2 and Fig. 5, these results demonstrate that the mitochondrial compartmentalization of Hsp22 is necessary for the migration of iNOS to mitochondria. In addition, these data show that the effect of Hsp22 on mitochondrial respiration requires an increase in mitochondrial localization of iNOS, but not necessarily a global increase in iNOS expression in the cell.

## Discussion

In this study, we demonstrate that the mitochondrial localization of Hsp22 mediated by its N-terminal domain is required for the stimulation of mitochondrial respiration via a mitochondrial iNOS-dependent mechanism.

The essential mitochondrial function of heat shock proteins has been demonstrated previously. For instance, it has been shown in optic lens that mitochondrial translocation of αB-crystallin preserves mitochondrial function during oxidative stress [24,25]. Mitochondrial and cytosolic Hsp70 play a vital role in mediating the translocation of nuclear-encoded proteins across the mitochondrial membranes and into the matrix, and facilitating their folding and assembly [26]. Mitochondrial Hsp10 and Hsp60 as well as Hsp70 are crucial for the folding and assembly of nuclear- and mitochondrial DNA-encoded subunits of the oxidative phosphorylation complexes [26]. Hsp10 and Hsp60 are mitochondria-specific and protect against apoptosis [27]. In *Drosophila*, mitochondrial Hsp22 increases longevity and promotes protection against oxidative stress [9]. Our own previous study has shown that deletion of Hsp22 impairs mitochondrial respiration [10] and results in a deterioration of cardiac function under stress.

Although location of Hsp22 in mitochondrial has been studied in Drospohila Melanogaster (DM) model[9], the sub mitochondrial distribution of Hsp22 in mammals has never been revealed. In this study, we identified for the first time that mitochondrial Hsp22 is predominantly associated with the inner membrane (IM) in the mammalian heart. The significance of this unique location of Hsp22 can be extrapolated from the critical role of the IM where oxidative phosphorylation is taken place to reform ATP through the molecules in the IM, implying a potential role of Hsp22 in mitochondrial respiration. To support this concept, we found that Hsp22 is also acts as an important regulator of mitochondrial genes which includes mitochondrial molecular carrier genes and heat shock proteins (Data not shown). One example from our results showed that overexpression of Hsp22 not only upregulates several of SLC25 family members, that are responsible for the molecular transport along the IM of mitochondria but also that Hsp22 showed a physically interaction with one of its members SLC25A25 in an isolated mitochondrial fraction extracted from the mouse heart (Data not shown). These data indicates a necessity of the physical location of Hsp22 location along the mitochondrial IM area. Our data is partially different from the finding in DM model in which Hsp22 predominantly located in mitochondrial matrix. One of the explanations for this difference may be partially due to the species difference and tissue specificity between two models. Mammals have a more comprehensive and sensitive aerobic metabolism than DM, accordingly require a different regulating mechanism. Also, the heart is one the most energy demanding tissues and totally dependent upon the oxidative phosphorylation chain to produce the large amounts of ATP required for the continuous contraction and relaxation.

In the present study, our first observation is that the mammalian Hsp22 translocates to the mitochondria of myocytes through its N-terminal domain. We show that N-terminal truncation of Hsp22 blocked its mitochondrial translocation, and impaired the regulation of oxidative phosphorylation.

Our second observation is that the mitochondrial transfer of Hsp22 is necessary for the well-known cytoprotective effects of Hsp22. Previously, we showed that hearts from the Hsp22 TG mouse are characterized by a major reduction in infarct size upon reversible coronary artery occlusion, and that such protection was lost after addition of the pan-NOS inhibitor L-NNA[5,7]. We showed that such cardioprotective effects also apply to isolated cardiomyocytes over-expressing Hsp22[12]. Accordingly, in the present study, when isolated myocytes were treated with a pro-apoptotic stimulus, Ad-WT-Hsp22 conferred a protective effect. However, overexpression of Ad-N20-Hsp22 resulted in a loss of this cardioprotective effect and an actual increase in apoptosis. Importantly, although the truncated N-terminal mutant Hsp22 protein impairs its subcellular localization in mitochondria, its effect on gene regulation in the nucleus was not affected, as shown by the sustained activation of the transcription factor STAT3. It is therefore likely that the cardioprotective effect of Hsp22 results from a synergy between gene regulation and mitochondrial localization.

Our third observation is that the mitochondrial localization of iNOS depends on mitochondrial translocation of Hsp22 itself, since overexpression of the Hsp22 N-terminal mutant, which fails to translocate to the mitochondria, increased iNOS expression in total protein but not in the mitochondrial compartment. These findings suggest the possibility of a co-translocation mechanism of Hsp22 and iNOS. Several studies have shown before that NOS is involved in mitochondrial physiology. The first report of the presence of a NOS isoform in the mitochondria was demonstrated by immunocytochemical localization of nitric oxide synthase in rat brain and liver mitochondria, which became known as mtNOS (mitochondrial nitric oxide synthase) [28–30]. In addition, NO stimulates mitochondrial biogenesis in cardiac muscle [31] through activation of several transcription factors, including PGC-1α, NRF-1 and the mitochondrial transcription factor A (TFAM).

Our fourth observation is that the mitochondrial transfer of iNOS and Hsp22 is necessary for the stimulatory effect of Hsp22 on mitochondrial respiration. Previously, we showed that increased Hsp22 expression in hearts from Hsp22 TG mice stimulates mitochondrial oxidative phosphorylation *in vivo*, whereas its deletion in a knockout model has the opposite effect [10]. Accordingly, in the present study, cardiac myocytes overexpressing Ad-WT-Hsp22 exhibited a significant increase in maximally stimulated mitochondrial respiration compared toβ-Gal treated cells, whereas overexpression of N20-Hsp22 did not increase the rate of oxidative phosphorylation, indicating that the role of Hsp22 in modulating the respiration of cardiac myocytes is dependent upon its localization in the mitochondria. In addition, the effect of Hsp22 on respiration was prevented upon iNOS inhibition, and the N-terminal Hsp22 mutant, which does not stimulate mitochondrial respiration, increased total iNOS expression in myocytes but failed to promote INOS translocation to the mitochondria. Taken together, these data demonstrate that the effect of Hsp22 on oxidative phosphorylation requires the mitochondrial translocation of both Hsp22 and iNOS. Importantly, although the truncated N-terminal mutant Hsp22 protein demonstrated an impaired mitochondria subcellular localization, its effect on gene regulation was not affected, as shown by the sustained activation of the transcription factor STAT3. Since STAT3 is known to promote iNOS gene expression [6,10], this may explain why, in the presence of Ad-N20-Hsp22, global iNOS abundance was still significantly increased although its mitochondrial translocation was impaired. It is therefore likely that the effect of Hsp22 on mitochondrial respiration results from a synergy between gene regulation and mitochondrial localization. However, the precise molecular mechanism controlling the interaction and importation of Hsp22 and iNOS in the mitochondria will require more studies at the subcellular level, since the main purpose of the present study was to elucidate the physiological consequence of this mechanism on mitochondrial respiration observed in our *in vivo* models.

The effects of NO on mitochondrial respiration are controversial. Some studies suggest that NO from either the endothelial or the inducible NOS causes decreased oxygen consumption *in vivo* [32]. For example, stimulation of the perfused rat heart with bradykinin or carbachol, which activate endothelial NOS, causes a decrease in oxygen consumption in cardiomyocytes [32,33], probably by inhibiting cytochrome oxidase. Increased iNOS levels have also been previously associated with reduction of mitochondrial respiration in pathological conditions, such as during hypoxia [34]. By contrast, other studies have shown that a moderate increase in iNOS expression in the heart, such as found during the second window of ischemic preconditioning or in our Hsp22 TG mouse model [5], prevents cell damage by promoting mitochondrial respiration [35]. There might be several reasons for such divergent results. First, most of the detrimental effects of iNOS on mitochondrial respiration were observed under stress conditions, especially when oxygen supply was low or interrupted [35]. In addition, iNOS exhibits variable tissue and cellular localizations. For example, under permanent coronary occlusion, iNOS increases predominantly in inflammatory cells but not in cardiac myocytes[35], while ischemic preconditioning increases iNOS in cardiac myocytes [35]. Therefore, depending on the model used, there can be a differential pattern of iNOS expression, as well as variable levels of NO production, which might explain the conflicting effects of iNOS on mitochondrial respiration. In our study, we clearly show that iNOS is necessary to promote mitochondrial respiration in a model of isolated myocytes, and, even more importantly, we determine that it is not the total cellular iNOS levels but rather the presence of iNOS in the mitochondria that is crucial for such stimulation. Increased mitochondrial translocation of iNOS by Hsp22, and the resulting enhanced mitochondrial respiration, may prove critical during cardiac stress. However, we also showed before [5] that Hsp22 blocks the mitochondrial pathway of apoptosis via caspase-9, Bad and Bcl_2_. It is therefore possible that the overall survival effect mediated by Hsp22 involves several mechanisms interacting inside the mitochondria.

Although other sources of NO production, such as eNOS, for example, may also be involved in the biological function of Hsp22, our investigation most likely associates specifically with the effects of iNOS, first because there is an excellent correlation between the mitochondrial translocation of iNOS and the observed physiological effects, and also because the inhibitor used in our study is acknowledged to be highly selective for iNOS [23]. Since NO production by iNOS is about 100-fold higher than that by eNOS [23], we are also confident that the nitrate assay reflected mainly iNOS-dependent NO production.

In conclusion, our study demonstrates a synergistic effect of Hsp22 and iNOS located inside the mitochondria in accelerating oxidative phosphorylation in cardiac stress, a condition during which the expression of both proteins is increased *in vivo*.
